# The role of potential agents in making spatial perspective taking social

**DOI:** 10.3389/fnhum.2013.00497

**Published:** 2013-09-05

**Authors:** Amy M. Clements-Stephens, Katarina Vasiljevic, Alexandra J. Murray, Amy L. Shelton

**Affiliations:** ^1^School of Education, Johns Hopkins UniversityBaltimore, MD, USA; ^2^Center for Talented Youth, Johns Hopkins UniversityBaltimore, MD, USA; ^3^Department of Psychological and Brain Sciences, Johns Hopkins UniversityBaltimore, MD, USA

**Keywords:** perspective taking, social skills, agency, individual differences, spatial cognition

## Abstract

A striking relationship between visual spatial perspective taking (VSPT) and social skills has been demonstrated for perspective-taking tasks in which the target of the imagined or inferred perspective is a potential agent, suggesting that the presence of a potential agent may create a social context for the seemingly spatial task of imagining a novel visual perspective. In a series of studies, we set out to investigate how and when a target might be viewed as sufficiently agent-like to incur a social influence on VSPT performance. By varying the perceptual and conceptual features that defined the targets as potential agents, we find that even something as simple as suggesting animacy for a simple wooden block may be sufficient. More critically, we found that experience with one potential agent influenced the performance with subsequent targets, either by inducing or eliminating the influence of social skills on VSPT performance. These carryover effects suggest that the relationship between social skills and VSPT performance is mediated by a complex relationship that includes the task, the target, and the context in which that target is perceived. These findings highlight potential problems that arise when identifying a task as belonging exclusively to a single cognitive domain and stress instead the highly interactive nature of cognitive domains and their susceptibility to cross-domain individual differences.

The ability to imagine the world from the point of view of another person comes in a variety of forms, from understanding another person's opinion on a discussion topic to literally imagining what the visual world would look like from their perspective. The latter, termed visual-spatial perspective taking (VSPT), has traditionally been considered a form of spatial problem solving. However, over the course of the last decade, there has been a growing body of research supporting a relationship between one's social abilities and the ease with which they are able to engage in this more visually driven form of perspective taking (Brunyé et al., 2012; Kessler and Wang, 2012; Shelton et al., 2012), highlighting the role of VSPT for everyday social interactions. Impairment on tasks that require adopting another's perspective—be it to judge if an object is visible from another viewpoint (Level 1 VSPT) or to represent what a spatial layout might look like from another viewpoint (Level 2 VSPT)—is a hallmark feature of Autism Spectrum Disorders (ASD; Baron-Cohen, 1992; Best et al., 2008). A vast majority of the research examining this relationship between social and VSPT abilities tends to come in two forms: either investigation of how/when VSPT abilities are impaired or preserved in individuals with ASD due to their known deficits in social skills (Hobson, 1984; David et al., 2006; Hamilton et al., 2009; Gould et al., 2011; Zwickel et al., 2011; Schilbach et al., 2012) or investigations of the natural variability that is observed in more typically-developing populations (Brunyé et al., 2012; Shelton et al., 2012).

One approach to understanding how social abilities might influence VSPT is through the investigation of the role that agents play in cognitive tasks. A wide range of literature on embodied cognition has investigated the conditions and tasks that appear to be sensitive to the presence of a human agent (Eppel et al., 1983; Schober, 1998; Ruby and Decety, 2001; Ames et al., 2008; David et al., 2008; Tversky and Hard, 2009; Kessler and Rutherford, 2010; Kessler and Thomson, 2010; Schilbach et al., 2012). For example, Schober (1998) has demonstrated that people will make an effort to adopt a listener's perspective when describing a spatial display, whereas they use their own perspective or neutral statements such as cardinal directions when asked to simply describe the display (no human listener). Similarly, Tversky and Hard (2009) asked individuals to describe spatial events depicted in scenes with or without the presence of another person in the scene. They found that the scene descriptions differed such that the participants spontaneously adopted the perspective of a person in a scene, even when such perspective taking was not relevant to the task. Moreover, they had no direct contact with the agent, suggesting that it was the mere presence and not any interactive requirement that motivated spontaneous perspective taking. Additionally, Schilbach et al. (2012) showed a sensitivity toward face-like stimuli that had a modulatory effect on performance when completing a gaze-mediated stimulus-response compatibility paradigm. When the social context of the stimuli was manipulated (face, face-like, or object stimulus), there was a reduction in the observed congruency effects for faces as compared to objects, suggesting that there was an effect of social context on action control. Based on these results, it is clear that human participants are sensitive to the presence of other human agents in ways that affect performance.

An alternative line of work has explored how agency might be attributed to objects (Zwickel, 2009; Zwickel et al., 2011; Zwickel and Müller, 2013). In a series of studies, Zwickel and colleagues set out to better understand VSPT when non-human entities were used as the target of perspective taking, both in typically-developing individuals (Zwickel, 2009; Zwickel and Müller, 2013) and those with ASD (Zwickel et al., 2011). This was first accomplished by examining whether individuals would adopt the perspective of geometrical shapes if the movement of the shapes appeared intentional. Intentionality was manipulated by using movements that implied interactions between the shapes. For example, when two triangles were moving about each other, they might evoke descriptions that reflect theory of mind (ToM) such as, “The small triangle surprised the large triangle.” Zwickel (2009) presented individuals with either the systematic ToM movement or random movement and found that individuals spontaneously adopted the perspective of the probed triangle when the movement implied agency but not when the movement was random. Follow-up work on individuals with ASD showed that although they were able to understand that the triangles were interacting in one case (ToM condition) and not in the other (random condition), the additional attribution of agency did not occur as evidenced by less appropriate descriptions of the animations (Zwickel et al., 2011). Therefore, it was concluded that in the case of individuals with ASD, although the perceptual cues (type of movement) were sufficient to invoke intentionality, they did not imbue the triangles with agency. These studies highlight one possible feature, intentional movement, which may be necessary for non-human targets to be perceived as potential agents. However, it is unknown what the potential boundary conditions are associated with perceived agency and the minimal requirements needed for stimuli to evoke VSPT when the stimuli are static.

In addition to understanding how agency attribution impacts VSPT, an additional line of research has been focused more on investigating the direct relationship between social abilities and VSPT. In particular, Brunyé et al. (2012) assessed whether gender and sub-clinical autistic traits were not only predictive of VSPT, but could differentiate between the levels of VSPT. The VSPT tasks used in this study required participants to either determine whether a light was visible from the perspective of an avatar (Level 1) or whether the light was to the left/right of the avatar (Level 2) and participants completed the autism quotient (AQ; Baron-Cohen et al., 2001). The overall score on the AQ was used with higher scores being indicative of greater autistic-like traits. Results from this study found slowed reaction times for the Level 2 VSPT task in males and females with relatively high AQ scores, suggesting that individuals with more autistic-like traits had greater difficulty taking the perspective of the avatar. This relationship was not seen for the Level 1 VSPT task. Taken together, these findings suggest that even in sub-clinical healthy populations, having more autistic-like traits influences one's ability to engage in perspective taking when the judgment to be made goes beyond asking whether something might be visible in the alternative perspective and requires a more complex set of judgments to be made about the spatial properties of the visual scene from an alternative perspective.

In a similar manner, Shelton et al. (2012) focused more specifically on social skills by using a combined score derived from the AQ social and communication subscales. As such, a lower score on this combined AQ score would be associated with individuals who have strong social skills whereas a higher score would be more associated with individuals who are less socially savvy^1^. In this experiment, participants were seated in front of a display of three buildings with seven different colored potential targets of perspective taking oriented around the display at 45° intervals. Participants were presented with an image and were asked to identify which viewpoint was being displayed, whether it be their own or one of the potential targets. Agency was manipulated by having each participant complete three different conditions: artist figures, triangles, and cameras. It was hypothesized that artist figures would be more human-like than either the triangles or cameras, with triangles as clear inanimate objects and cameras as potential intermediaries of perspective (people look through them). A striking relationship was found. Participants with lower AQ combo scores (more social) were more accurate at taking the perspective of the artist figures (*r* = −0.584) than those with higher AQ combo scores (less social), whereas no such relationship was found for either the triangles (*r* = −0.084) or the cameras (*r* = −0.053) conditions. It should be acknowledged that all of the conditions used objects, but the relative amount of potential agency conveyed varied across the different conditions. As such, these findings point to another potential requirement for perceived agency, especially with respect to static images; that is, it may be necessary for the potential “agents” to possess some human-like qualities.

Not only do these studies provide indications as to what it means for an object to be perceived as a potential agent, they also introduce a framework for distinguishing when VSPT includes a social component or not. That is, VSPT may be primarily spatial and remain so when targets do not evoke the suggestion of social engagement, but VSPT may become more dependent on interactions with social skills when targets are more agent-like, allowing one's social skills to influence behavior for better or for worse. This offers a method for assessing what kinds of targets might make VSPT more or less social. In particular, we can use the correlation between VSPT performance and social skills as a measure of when VSPT is or is not incurring social skill influence. If a target is motivating the task to be a “socially relevant” form of VSPT, we expect a relationship between measures of social skill and VSPT performance. However, if a target is not incurring the agency necessary to motivate social relevance, we expect to see a “non-social” form of VSPT such that social skills are not correlated with VSPT performance. Critically, we do not predict opposite relationships for socially relevant and non-social VSPT, but rather suggest that the task can either be sensitive to social influence or not (see Figure 1).

**Figure 1:**
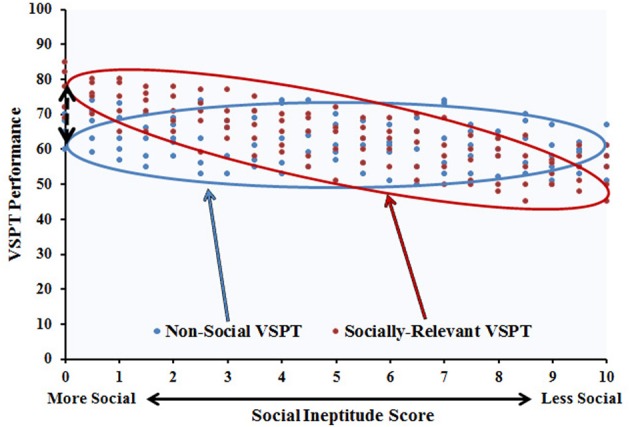
**Potential framework depicting the relationship between social skills and VSPT performance for proposed socially-relevant and non-social VSPT**. The distinction between these two types of tasks is captured by the degree to which VSPT is incurring a social skill influence indexed by the magnitude of the correlation.

Using this approach to assess social influence, we can begin to ask deeper questions about what targets, target features, and conditions change the way an individual approaches the VSPT task. First, we can test how changing basic features might influence the degree to which a target seems to acquire agency that brings social skills to bear. In the previous study (Shelton et al., 2012), we used brute force differences (human form vs. inanimate objects), but more subtle information can also be manipulated. For example, we can manipulate the presence or absence of very basic facial features. Second, we can ask whether and how experience with one type of target might modulate the perceived agency of another target, which we refer to as experiential context. In the previous study (Shelton et al., 2012), there was no effect of order, suggesting that having seen the artist figures first did not make the triangles more or less sensitive to the influence of social skills. Moreover, seeing the triangles followed by artist figures did not make the artist figures more or less sensitive. However, as noted above, these were already different classes of objects. Here we consider what happens to perceived agency when targets share certain features but not others.

To address these issues, we ran a series of experiments that compare the original targets from the previous study, plain triangles and artist figures, to other variations that might convey more or less agency as evidenced by an influence of social skills on VSPT performance. First, we set out to establish whether adding human-like features to an object would increase the sensitivity to social skill influence. In Experiment 1, we compared plain triangles to triangles with eyes affixed to them, making them appear more human-like (or at least Muppet-like) via visual features. In Experiment 2, we compared the artist figures condition from our previous study, which showed the relationship between social skills and VSPT, to agents with even more human-like qualities, fashion dolls. In both Experiments 1 and 2, we contrasted conditions that vary on known visual features and counterbalanced order to allow us to explore any modulation of the social skill relationships due to experiential context. Lastly, we wanted to ask whether we could increase sensitivity to social skill influence by conceptually manipulating the meaning of a target of perspective taking. In Experiment 3, we return to the plain triangles, but now refer to them as “Aliens” in an effort to convey that these could be creatures with agent-like qualities. This manipulation allowed us to ask whether a conceptual cue to agency can be robust enough to bring about the influence of social skills on VSPT performance. Overall, results from these studies reveal the complexity of the relationship between social skills and VSPT, highlighting the susceptibility of individual differences to contextual and cross-domain influences.

## General materials and methods

All three of the correlational experiments used the same basic paradigm, varying only the target of the VSPT task.

### Participants

All participants were Johns Hopkins University undergraduate students between the ages of 18–22 who participated in return for extra credit in psychology courses. All procedures were approved and conducted in accordance with the Johns Hopkins Homewood Institutional Review Board. For all studies, inclusion in the study was based on the 0° orientation trials (described in more detail in the subsequent section). Because this type of VSPT task is difficult and a wide range of scores is typically observed, we did not want to exclude individuals merely because they fell along the lower end of the distribution. Therefore, we reasoned that if participants could correctly identify their own view, then we could assume that they understood and were engaged in the task. As such, we excluded individuals who made more than one error on these trials. Across all of the experiments, this criterion seemed to successfully separate those who were on task from those who were not. Moreover, for each experiment, an effort was made to obtain approximately equal numbers of males and females. The analyses for each experiment included examining potential differences in performance between the genders. Consistent with previous findings obtained by Shelton et al. (2012), the differences between males and females on all measures and correlations were negligible and will not be discussed further.

### Materials, design, and procedures

For each experiment, participants completed a set of measures that included the three buildings task, paper-and-pencil spatial skill tests, and a self-report questionnaire (the AQ described below) in a pseudorandom order. The spatial skill tests administered are part of a standard battery of measures typically included across all experiments in the lab; they were not pertinent to the hypothesis-driven questions being addressed and had little or no relationship to the outcomes presented below.

#### Three buildings (3Bldgs) task

**Figure 2:**
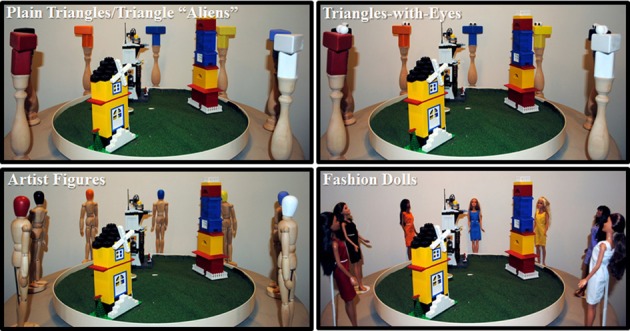
**Example of one of the three buildings displays showing the different target conditions used in the Experiments: upper left (Experiments 1 and 3), upper right (Experiment 1), lower left and right (Experiment 2)**.

Participants completed the 3Bldgs task, which is an adaptation of Piaget's three mountains perspective-taking test (Piaget and Inhelder, 1967). For this task, participants viewed two different displays. Each display consisted of three unique buildings (6 different buildings total) with each building constructed out of LEGO® building blocks (Lego Group, Billund, Denmark) and placed on 24″ diameter plastic disks that were covered in faux grass mats. Each display disk was centered on a 36″ diameter wood table and photographed from 8 different orientations separated by 45° increments. Around the building display were seven uniquely colored targets for perspective taking (red, blue, white, black, purple, yellow, and orange). Targets were placed at 45° intervals and corresponded to headings of 45°, 90°, 135°, 180°, 225°, 270°, and 315° with respect to the participants' designated view of 0°. The targets were manipulated across the set of experiments to assess the potential impact on the perceived agency (see Figure 2) and are described in greater detail with the corresponding experimental manipulation.

Participants were seated in front of the physical display and viewed images on a laptop computer. Each presented image corresponded to the would-be visual perspective of one of the targets or to the participant's own perspective (0°). Participants were asked to identify the perspective of the image. For each image, irrespective of the task version, the participant was asked, “Which <TARGET> is at this view?^2^ ” Participants indicated their response by pressing a key corresponding to the color of the target or the spacebar to indicate that it was his/her own view. Each task version consisted of 40 self-paced trials (5 trials at each orientation) with a 5-s response deadline. Response latency and accuracy were measured.

The 0° orientation (where the participant was seated) was selected randomly for each participant from one of 4 possible orientations. For each display, the four candidate orientations were selected by randomly choosing one orientation and using the 3 additional orientations that were opposite and orthogonal to that initial orientation. For a given start orientation, targets were placed at the remaining seven orientations. When appropriate, display-condition assignment and order of conditions were counterbalanced, and the order of the target colors around the display was selected randomly for each participant and kept constant for both conditions (when applicable).

#### Autism quotient (AQ; Baron-Cohen et al., 2001)

The AQ is a self-report questionnaire designed to assess the degree to which individuals vary on five traits typically associated with ASD—social skills, perseveration, attention to detail, communication, and imagination—with higher scores (overall and each subscale separately) reflecting stronger ASD-like traits. For the set of experiments presented here, the critical scales of interest are the social and communication impairment scales, which are designed to capture behaviors on a continuum from socially appropriate to socially inappropriate behaviors. Due to the strong correlation observed in Shelton et al. (2012) between the social and communication impairment subscales, we used the same combined social/communication score as the previous study. For clarity, we term this the social ineptitude score, reflecting the fact that higher scores mean less social. This social ineptitude score is used in all analyses for each experiment.

## Experiment 1: triangles with and without eyes

In this experiment, we set out to assess whether objects could be made sensitive to the influence of social skills by adding features suggestive of agency. Specifically, participants were asked to complete two conditions in which they either took the perspective of a triangle (plain triangles condition) or took the perspective of a triangle that had eyeballs affixed to the top of it (triangles-with-eyes condition). First, we expected no relationship between social skills and performance with plain triangles, replicating our previous work. For the triangles-with-eyes condition, we used the magnitude of the correlation between social skills and performance as an index to determine whether eyeballs were sufficient for inducing potential agency in an object. If any sign of agency “socializes” the task, then we might expect a correlation comparable to that observed for the artist figures in Shelton et al. (2012). However, we may also see a gradation of social skill influence dependent upon the degree of potential agency induced, in which case the triangles-with-eyes would show a weaker correlation than other more agent-like targets. Such a result would necessitate additional comparisons. Finally, we might see that static triangles are objects regardless of whether they have eyes or not, with no correlation observed in either condition.

In addition to the basic comparison of triangles with and without eyes, we also entertained the possibility that the order of the conditions could affect observed correlations. Given that these two conditions use the same basic object, they may provide a more direct contrast of their potential agency than the triangles, cameras, and artist figures used in our previous research study (Shelton et al., 2012). As such, we considered whether seeing the plain triangles first might imbue the subsequent triangles with eyes with more agency than they might convey on their own (when experienced first). This might result in the correlation for triangles-with-eyes being weaker when performed first than when performed after plain triangles. Plain triangles provide an even more interesting case in that they are expected to show no correlation, especially if experienced first. However, it is possible that seeing triangles-with-eyes as potential agents could carryover to subsequent performance with plain triangles, making them more sensitive to the social skill influence.

### Materials and methods

#### Participants

For this experiment, 78 naïve participants were enrolled. Six participants (2 males) failed to meet criterion, leaving 72 participants (34 males) included in all subsequent analyses.

#### Materials, design, and procedures

Participants completed two versions of the 3Bldgs task using plain triangles and triangles with eyes as targets. Using a set of 14 identical wooden triangular blocks, we created two sets of seven different colored triangles (see above) placed on plain wood pedestals (13″ total height). One set served as the plain triangles condition. For the second set, 1″ round wooden eyeballs painted white with black circles were affixed to the top of each triangle to create the triangles-with-eyes condition (see Figure 2). For each image, irrespective of the task version (plain triangles/triangles-with-eyes), the participant was asked, “Which Triangle is at this view?” Each participant completed both versions of the task using two different displays. Display-condition assignment and order of conditions were counterbalanced. After applying the exclusion criterion, we had approximately equal numbers in each order (plain triangles first *n* = 34). The order of the target colors around the display was selected randomly for each participant and kept constant for both conditions.

### Results

**Figure 3:**
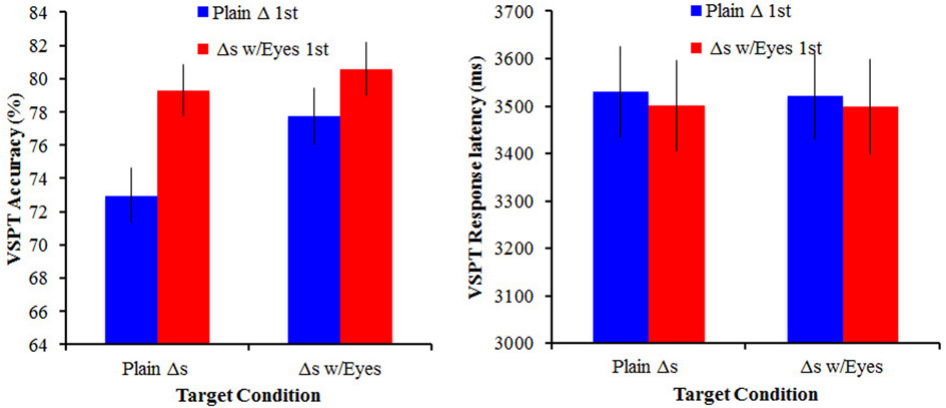
**VSPT performance (mean accuracy and response latency) as a function of the target conditions for Experiment 1**. Error bars reflect ±1 standard error of the mean.

Mean response latency (overall and for correct trials only) and overall accuracy were calculated for both versions of the 3Bldgs task and were separately subjected to a mixed ANOVA with order as a between-subjects variable and target (plain triangles/triangles-with-eyes) as a within-subjects variable (Figure 3). For response latency, there were no significant effects or interactions (all *p*s > 0.11). For accuracy, we found that the group that performed the plain triangles first were significantly less accurate than the group that performed the triangles-with-eyes condition first, *F*_(1, 70)_ = 5.75, *p* = 0.019, η^2^_G_ = 0.05. Although this effect was significant, it was a small effect, accounting for only about 5% of the measured variance. Moreover, we also observed that participants were significantly more accurate on the triangles-with-eyes condition than the plain triangles condition, *F*_(1, 70)_ = 5.98, *p* = 0.017, η^2^_G_ = 0.02. Again, this was a small effect (2% of the measured variance). There was no significant order × target interaction, *F*_(1, 70)_ = 1.97, *p* = 0.165. Although these effects are fairly small, it suggests that it may be important to consider whether some targets' perspectives are more readily adopted overall, and, more critically, the role order may play when investigating the relationship between social skills and performance on our VSPT conditions.

In addition to assessing conditional differences, we also correlated the performance on the plain triangles and triangles-with-eyes conditions. Accuracy was positively correlated, *r* = + 0.44, *p* < 0.001. This relationship is consistent with our previous study (Shelton et al., 2012) and suggests that there is a common spatial component to the VSPT task, irrespective of target condition.

To answer the critical question of social skill influence on VSPT performance we explored the correlations between social skills and accuracy^3^ on the VSPT task for the two target conditions overall and then for each order separately. All correlations are significant at α = 0.05, corrected for the size of the relevant subset of correlations investigated unless otherwise specified. Consistent with Shelton et al. (2012), we observed a significant correlation between the social and communication impairment subscales from the AQ (*r* = 0.42); therefore, due to this observed relationship and to be consistent with previous literature we used a combined scored (social ineptitude score) in all analyses (see Table 1 for separate correlations with subscales). Lower values on the social ineptitude score would be associated with better social skills, whereas higher values on the social ineptitude score would be associated with poorer social skills. Overall, we observed no correlation for the plain triangles condition, *r* = −0.18, *p* = 0.12 and a negative correlation for the triangles-with-eyes condition, *r* = −0.46, indicating that more social individuals had better performance than less social individuals for the triangles-with-eyes condition. A *t*-test for non-independent *r*'s was conducted and revealed that these correlations were significantly different, *t*_(69)_ = 2.45, *p* = 0.02, suggesting that adding eyes to the plain triangles allowed objects to become sensitive to social skill influences in VSPT.

**Table 1:** **Summary of correlations (and *p*-values) between the relevant social skill scores and each condition, overall and by order of conditions where relevant**.

	**Social subscale**	**Communication subscale**	**Social ineptitude score**
**EXPERIMENT 1**			
**Plain triangles**			
Overall	+0.01 (0.939)	−0.33 (0.005)^*^	−0.18 (0.123)
1^st^	+0.23 (0.202)	−0.04 (0.805)	+0.13 (0.479)
2^nd^	−0.20 (0.232)	−0.50 (0.001)^*^	−0.43 (0.008)^*^
**Triangles-with-eyes**			
Overall	−0.26 (0.031)	−0.54 (<0.001)^*^	−0.46 (<0.001)^*^
1^st^	−0.29 (0.077)	−0.46 (0.003)^*^	−0.46 (0.004)^*^
2^nd^	−0.22 (0.219)	−0.60 (<0.001)^*^	−0.45 (0.008)^*^
**EXPERIMENT 2**			
**Artist figures**			
Overall	+0.01 (0.920)	−0.15 (0.195)	−0.08 (0.521)
1^st^	−0.25 (0.160)	−0.45 (0.007)^*^	−0.39 (0.022)
2^nd^	+0.20 (0.235)	+0.07 (0.678)	+0.14 (0.391)
**Fashion dolls**			
Overall	−0.25 (0.032)	−0.48 (<0.001)^*^	−0.40 (0.001)^*^
1^st^	−0.21 (0.209)	−0.41 (0.011)	−0.33 (0.045)
2^nd^	−0.32 (0.067)	−0.61 (<0.001)^*^	−0.52 (0.002)^*^
**EXPERIMENT 3**			
Triangle aliens	−0.27 (0.066)	−0.34 (0.017)^*^	−0.36 (0.013)^*^

All p-values presented are uncorrected for multiple comparisons;

* indicates the correlations that survive the correction for multiple comparisons within related subsets.

**Figure 4:**
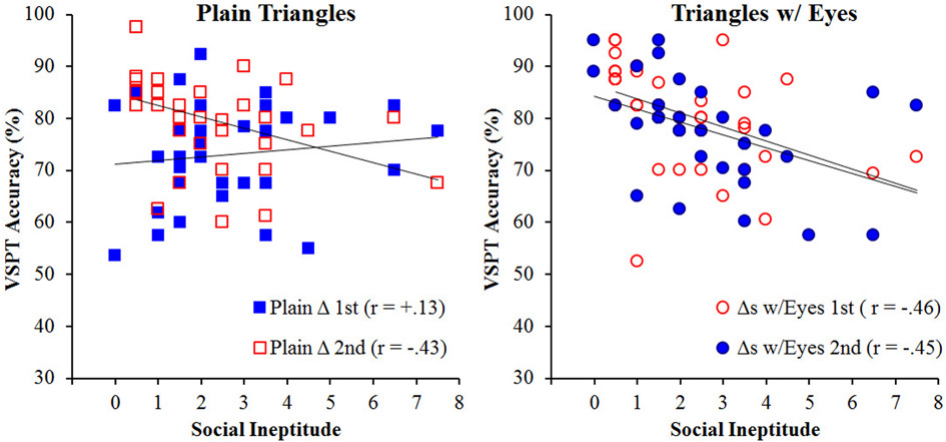
**VSPT performance as a function of social ineptitude score separately for the plain triangles (left) and triangles-with-eyes (right) conditions broken out by order for Experiment 1**.

An examination of the effect of order on these correlations paint a more complex picture, as can be observed in Figure 4, which shows the correlations for both conditions separately for each order. For the triangles-with-eyes condition, the correlation was significant regardless of the order in which the conditions were completed, *r* = −0.46 and −0.45, supporting the claim that placing eyes on the simple triangles was sufficient to bring about the social skill influence. Performance on perspective taking with plain triangles had previously not shown a significant correlation with social skills (Shelton et al., 2012), and that was again the case when this condition was performed as the first condition, *r* = +0.13, *p* = 0.48. However, when the plain triangles followed the triangles-with-eyes, there was a significant negative correlation, *r* = −0.43, that was not significantly different from those observed for the triangles-with-eyes in either order, *p*s > 0.45. Additionally, this correlation was significantly different from the correlation when the plain triangles were viewed first as evidenced by a *z*-transform for independent correlations, *z* = −2.35, *p* = 0.009. As such, plain triangles incurred as much influence from social skills as triangles-with-eyes when participants had experienced the triangles-with-eyes first.

In cases where we observed a correlation with the social ineptitude score, we also compared their magnitude to the correlation observed for artist figures in the previous study (*r* = −0.58; Shelton et al., 2012) using a *z*-transform for independent correlations. None of the comparisons were significant, all *ps* > 0.16, suggesting that the triangles-with-eyes and the plain triangles (when presented after the triangles-with-eyes condition) were showing correlations in the same range as previously observed for artist figures.

### Discussion

The goal of Experiment 1 was to determine whether an object previously shown to be insensitive to social skill influence could be made sensitive by adding an agent-like feature. Adding eyes to triangles had two important impacts on performance. First, we did observe the significant correlation between performance on the triangles-with-eyes condition and social skills, suggesting that features such as eyes can create targets of perspective taking that are sensitive to the influence of social skills. In essence, it appears placing static features on an object may convey a sense of agency similar to what was observed for animating shapes (e.g., Zwickel, 2009). In addition, we observed that our plain triangles could also be imbued with some agent-like attributions by simply following the experience of the task with the triangles-with-eyes.

It is tempting to argue that the act of performing the task with an implied social context might keep participants in a state of “social-ness” rather than actually imparting the agency or sociality on the plain triangles. This seems unlikely given that we failed to find any order effects (or even trends that would suggest order effects) in the previous study (Shelton et al., 2012) and pilot work when the targets were different kinds of objects. Instead, it seems that the shared properties of the triangles with and without eyes may have allowed the agency (or sensitivity to social influence) induced by the eyes to carry over. In Experiment 2, we examine a variation on this carry over effect by contrasting two different representations of human form.

## Experiment 2: artist figures and fashion dolls

Experiment 1 started with an object and examined whether we could induce sensitivity to social skill influence. In Experiment 2, we started with the artist figures that were first used to demonstrate the correlation between social skills and VSPT in this paradigm and compared them to a target with more human-like features to assess whether the correlation might be sensitive to the degree or extent of implied agency. In addition, we again varied the order of the conditions to examine whether experience with the putatively stronger potential agent might strengthen or weaken the sensitivity of the putatively weaker potential agent.

### Materials and methods

#### Participants

For this experiment, 82 naïve participants were enrolled. Ten participants (5 males) were excluded due to failure to reach criterion, leaving 72 participants (30 males) eligible for all subsequent analyses.

#### Materials, design, and procedures

Participants completed two versions of the 3Bldgs task. In the artist figures condition, each target was a 13″ tall wooden artist figure with its head painted one of seven unique colors. In the fashion dolls condition, we used a set of 7 distinct Barbie™ dolls (Mattel, Inc., El Segundo, CA), with each fashion doll wearing a colored dress corresponding to the colors used in the artist figures condition (see Figure 2). For each image, irrespective of the task version (artist figures/fashion dolls), the participant was asked, “Which Doll is at this view?” Each participant completed both versions of the task using two different displays. Display-condition assignment and order of conditions were counterbalanced. After applying the exclusion criterion, we had approximately equal numbers in each order (artist figures first *n* = 38). The order of the target colors around the display was selected randomly for each participant and kept constant for both conditions.

### Results

**Figure 5:**
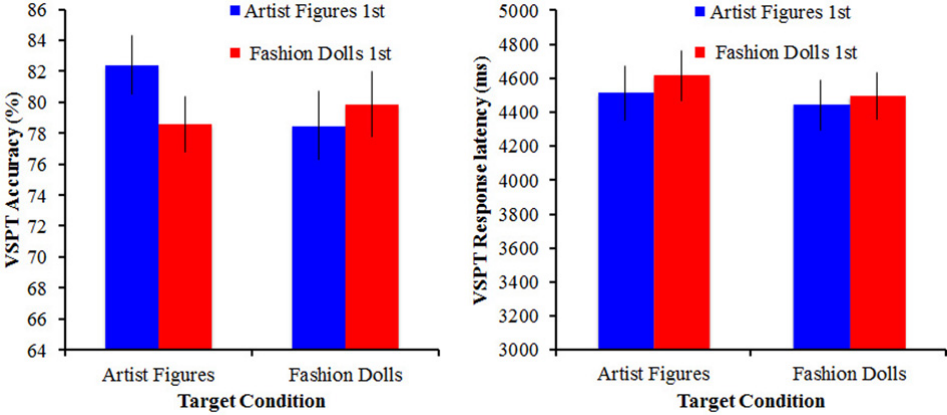
**VSPT performance (mean accuracy and response latency) as a function of the target conditions for Experiment 2**. Error bars reflect ±1 standard error of the mean.

Mean response latency (overall and for correct trials only) and overall accuracy were calculated for both versions of the 3Bldgs task and were separately subjected to a mixed ANOVA with order as a between-subjects variable and target (artist figures/fashion dolls) as a within-subjects variable (see Figure 5). For both response latency and accuracy, there were no significant effects or interactions (all *p*s > 0.23 for response latency and all *p*s > 0.07 for accuracy). Again, we observed a significant positive correlation between accuracy with fashion dolls and accuracy with artist figures, *r* = +0.46, *p* < 0.001.

Given the observed correlation between the social and communication impairment subscales from the AQ (*r* = 0.69), all subsequent correlations were run between the social ineptitude score and the accuracy on the two agency conditions overall and then for each order separately (see Table 1 for separate correlations with subscales). All correlations are significant at α = 0.05, corrected for the size of the relevant subset of correlations investigated unless otherwise specified. Overall, we observed a negative correlation for the fashion dolls, *r* = −0.40, indicating better accuracy with better social skills. Surprisingly, no such correlation was observed for the artist figures, *r* = −0.08, *p* = 0.52. This is contrary to our previous studies where we have consistently observed this correlation (Shelton et al., 2012). A *t*-test for non-independent *r*'s confirmed that these two correlations were significantly different from each other, *t*_(69)_ = 2.82, *p* = 0.006.

**Figure 6:**
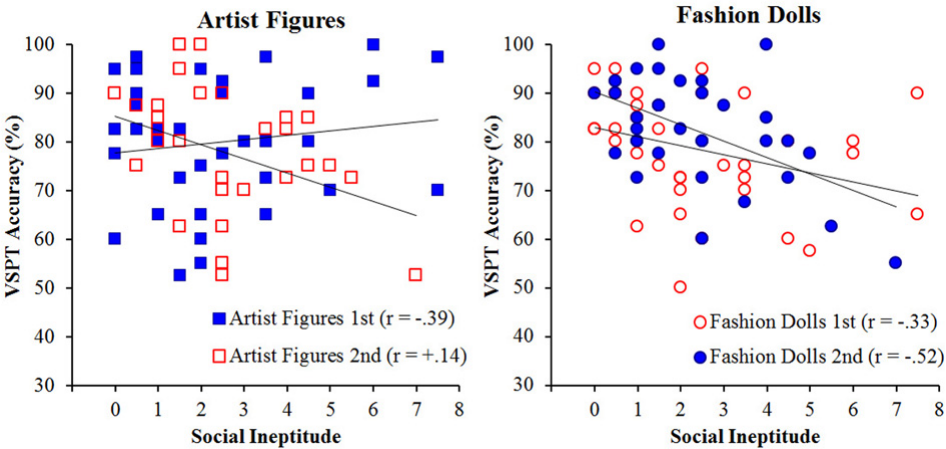
**VSPT performance as a function of social ineptitude score separately for the artist figures (left) and fashion dolls (right) conditions broken out by order for Experiment 2**.

The unexpected result in the artist figures overall made the motivation for examining order effects even stronger. Figure 6 shows the correlations broken down by order. For the fashion dolls, the correlation between social ineptitude score and performance was weaker when fashion dolls were presented first, *r* = −0.33, *p* = 0.04 uncorrected, but met the criterion for multiple comparisons when fashion dolls were presented second, *r* = −0.52. Although the correlation numerically increased when fashion dolls were presented second, the difference between the two correlations was not significant, *z* = 0.97, *p* = 0.17, suggesting that the correlation was similar irrespective of order. An examination of the artist figures condition revealed a more complicated picture. That is, when artist figures came first, performance showed a correlation with the social ineptitude score similar to what was observed previously (Shelton et al., 2012), *r* = −0.39, *p* = 0.02 uncorrected (comparison to *r* = −0.58, *z* = 1.33, *p* = 0.09), but when these same artist figures followed the experience with fashion dolls, the correlation with the social ineptitude score was weak and in the opposite direction, *r* = +0.14, *p* = 0.39. The correlations for the artist figures in the two different orders were significantly different, *z* = 2.26, *p* = 0.001, suggesting that the social skill sensitivity was modulated by the context in which the particular targets were experienced.

### Discussion

Experiment 2 provides a second example of how the degree to which a target of perspective taking appears to be socially relevant can be influenced by the experience of other targets. Artist figures, which were used to establish the initial correlation between social skills and VSPT in this paradigm, were essentially stripped of their sensitivity to social skill influence when they were experienced after performing the task with fashion dolls.

Although both Experiments 1 and 2 demonstrate how context can affect the sensitivity to social skill influences, the results may seem contradictory. In Experiment 1, having the putatively more agent-like target first increased the sensitivity for the subsequent less agent-like target, whereas Experiment 2 showed the opposite effect. However, the figures used in Experiment 2 were not the same object varying in a feature or two; they were two different representations of human form that varied on a variety of visual features (continuity of form, faces, hair, clothing, etc.). Although the artist figures can clearly convey agency in a way that allows a social skill influence, they are also affected by the context in which they are experienced.

The broader issue of context effects and target influences will be addressed in more detail in the General Discussion, but first we turn our attention to another form of context. All of our manipulations of potential agent-like features so far have been visual features. It is also possible to create conditions in which objects might be viewed as agents via conceptual context. Whether we can induce sensitivity to social skill influence using a conceptual context is the question for Experiment 3.

## Experiment 3: triangle “aliens”

One of the clear conclusions of Experiments 1 and 2 is that objects can be sensitive to social skill influences as a function of having features that suggest potential agency. Whether affixing eyes to simple shapes or using representations of the human form, these features appear to affect how individuals approach the perspective-taking task. We also observed a form of conceptual carryover from the triangles-with-eyes to the plain triangles. As a final test, we asked whether a purely conceptual manipulation could also make an object sensitive to social skill influence. Using the plain triangles again, we offered an alternative interpretation of the triangles as potential agents by calling them “aliens” during the perspective-taking trials. If triangles with eyeballs on top motivate the task to become more social in nature, then perhaps simply suggesting a type of being, be it alien or otherwise, might operate in a similar manner.

### Materials and methods

#### Participants

For this experiment, 53 naïve participants were enrolled. Five participants (3 males) failed to meet criterion, leaving 48 participants (24 males) included in all analyses.

#### Materials, design, and procedures

Participants completed the 3Bldgs task using the same 7 triangular blocks on pedestals described in Experiment 1. For each image the participant was asked, “Which Alien is at this view?” Across participants, the display type was counterbalanced and the order of the target colors around the display was selected randomly for each participant.

### Results and discussion

**Figure 7:**
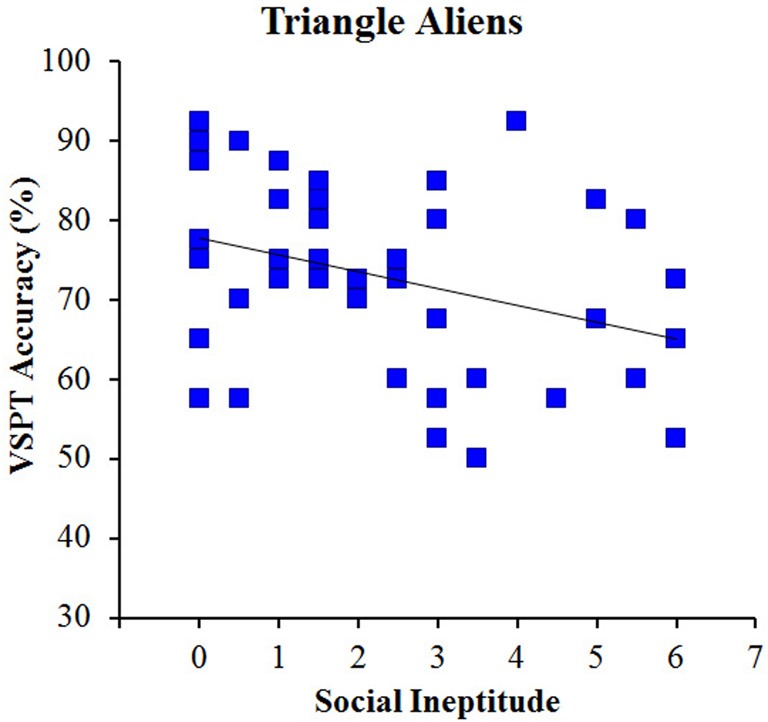
**VSPT performance as a function of social ineptitude score for the triangle aliens condition**.

Mean response latency was 3018 and 3054 ms overall and for correct trials only, respectively, and overall accuracy was 72.9%. Again, we observed a significant correlation between the AQ social and communication impairment subscales, *r* = +0.46, so we again used the social ineptitude score (see Table 1 for separate correlations with subscales). The critical correlation between the social ineptitude score and performance on the VSPT task with triangle aliens was significant, *r* = −0.36 (see Figure 7). Moreover, this correlation was not significantly different from the correlation obtained for the triangles-with-eyes condition either overall or separated by order in Experiment 1, *p*s > 0.65. These results suggest that even in the absence of a visual feature, an object can become sensitive to social skill influence on VSPT through conceptual suggestion.

## General discussion

One of the key motivations for this special issue on developing a framework for integrating the “social” and the “spatial” is the recent acknowledgment of a clear relationship between VSPT and aspects of ones savvy in social situations (Hamilton et al., 2009; Zwickel et al., 2011; Brunyé et al., 2012; Shelton et al., 2012). By definition, VSPT tasks involve the need to consider/imagine/reason about a target perspective that is different from one's own. As such, it is tempting to conclude that VSPT is both a spatial and social task. However, these tasks can vary dramatically with respect to who or what is the placeholder for the target perspective. Our previous work has shown that the relationship between VSPT performance and social skills depends on the nature of the target; targets that could be seen as potential agents were sensitive to social skill influence, whereas targets that did not have agent-like features were insensitive (Shelton et al., 2012). These and similar results (Zwickel, 2009; Zwickel et al., 2011; Schilbach et al., 2012; Zwickel and Müller, 2013) suggest that VSPT tasks *acquire* some social relevance when the target has potential agency. Using correlations between measures of social skills and performance on VSPT, the present study offers a more detailed account of what features and conditions can affect the degree to which any given target might convey agency and become sensitive to social skill influence.

One of the first implications of the present work is that simple physical features can induce an object to be sensitive to social skill influence. In the previous study, the physical form of a wooden artist figure appeared to motivate participants to engage the VSPT task in a more social manner than either plain triangles or cameras (Shelton et al., 2012). Although triangles do not resemble human forms, in the present study, we were able to observe the same social skill influence on VSPT by simply adding eyeballs to the triangles. This small featural change seemed to push participants to engage the triangles as if they were potential agents, like the artist figures. Taken together, this body of work suggests that there are different visual features that can motivate one to perceive an object as a potential agent. The global shape of the artist figures likely conveyed the sense that this could be a person form, whereas the presence of eyes (which the artists figure did not actually have) likely conveyed a similar sense for triangles. These differences raise important questions about what other features or combinations might be more or less effective in engaging the social mechanisms that appear to be brought to bear, but they establish the very basic notion that minimal change can assert a strong influence.

In addition to the observation that physical features can induce agency, we also observed that the context defined by experiences surrounding the introduction of particular targets in perspective taking can also play a role in the perceived potential agency. First, we found that when a target closely resembles another object that has recently been attributed with agency, the similarity of object features may be sufficient to convey carryover agency. Performing VSPT with plain triangles as targets is generally insensitive to social skill influence when performed prior to other conditions or in the context of artist figures or cameras. That is, on their own, they are not potential agents. Despite their fundamental characterization as inanimate objects, these same plain triangles can be engaged as if they were potential agents when they immediately follow experience with identical triangles with eyes affixed to them. In other words, experiencing triangles-with-eyes as agents allowed plain triangles to be viewed as agents.

The artist figures provide a second case of experiential context. When performing the task with artist figures in the context of plain triangles or cameras, the artist figures represent the condition that shows the strongest numerical relationship between social skills and performance (Shelton et al., 2012). Similarly, when participants experienced the task with the artist figures first in Experiment 2, we saw a similar performance-social skill correlation to other “agent” conditions. However, when participants experienced the artist figures after exposure to the fashion dolls, this correlation was diminished completely for the artist figures condition, such that it had the same magnitude correlation as plain triangles alone. In this case, it was as if experiencing the fashion dolls as agents made the artist figures seem not only *less* agent-like but not at all agent-like, akin to a purely inanimate object.

As noted previously, the two types of triangles and two types of dolls represent opposing effects of experiential context (for a more in-depth discussion of experiential social context or historicity, see Schilbach et al., 2013). For the triangles, we had identical objects that differed only in the presence or absence of a single agent-like feature (eyes). Whatever attributes the eyes appeared to bring to the triangles on which they were affixed (triangles-with-eyes condition) lingered when the triangles were presented again without eyes (plain triangles). One argument might be that the triangles were viewed as the same triangles with and without eyes. That is, once the triangles had been imbued with potential agency, having them “return” without eyes did not immediately strip them of the attributes that were motivating the task to take on social relevance. By contrast, the fashion dolls and artists figures are clearly not the same object but are different representations of human form. Although artist figures alone may be sufficient to engage the social mechanisms that affect performance with agent-like targets, they appear to lose any attributes that convey a sense of agency when one has had experience with the more representative form of the fashion doll. Anecdotally, some participants even remarked that the artist figures seemed “creepy” after seeing the fashion dolls. This type of comment has never been noted before in our previous studies nor in the condition when the artist figures came first, suggesting that the experiential context was asserting a strong influence over the perception of the objects at a very basic level.

Although not significantly different from the cases presented here, it is notable that the artist figures in Shelton et al. (2012) had a numerically larger correlation than any of the conditions in the present study. This may be due to the fact that the artist figures were experienced in the context of two other objects that did not possess agent-like qualities (plain triangles and cameras). In 2/3 of the orders used for the study, the artist figures would have come after one or both of the object conditions, which may have magnified the correlation relative to the conditions used in the present study. The lack of order effects on the correlations in the original study is potentially problematic for this argument. However, given that the artist figures when presented first had a correlation of −0.39 in the present study it is possible that the original study was insufficiently powered to detect changes from such a high baseline correlation. Again, this suggests that the role of experiential context may be a critical factor in the way individuals engage a VSPT task with different targets.

Both the induction of agency and contextual effects noted above are driven by physical features. For example, the carryover of agency from triangles-with-eyes to plain triangles likely depended on the agency evoked by the physical features of the eyes carrying over to the highly similar plain triangles. Likewise, the many agent-like features of the fashion dolls appeared to convey agency, and the diminished agency for artist figures following experience with fashion dolls likely depended on the contrast of these “rich” agents to the highly dissimilar artist figures. However, the conveyance of agency is not limited to physical features. In Experiment 3, the performance on the VSPT task with plain triangles was again correlated with social skills when the triangles were referred to as aliens rather than triangles. Implying a being, albeit an alien being, appears to be similar to adding physical features such as eyeballs, motivating participants to engage in the task in a way that allows social skills to assert influence. This suggests that when interpreting the targets of a VSPT task, people may have a very low threshold for allowing a target to be “social.”

A running theme through this work is the introductory framework in which the target and possibly other features of a VSPT task can affect whether the task itself is socially relevant or not. Using this framework, we have demonstrated that the degree to which a task appears to be social or not involves the complex interaction of target features and experiential context, which includes the presence of other targets and the language used to identify the targets. One might be tempted to conclude that this work is largely about methodology, and in some sense, this is the case. Although we have not exhaustively tested all types of targets or target combinations, our findings clearly offer some suggestions for how to craft a VSPT task that is or is not sensitive to social skill influence. For example, if one's goal is to understand VSPT in isolation, what we have termed non-social VSPT, then one can design a task that limits the potential agency as much as possible. However, this work also speaks to and raises many deeper theoretical issues about how the human brain processes spatial information in order to reason about the world.

A first critical point is that the sensitivity of VSPT performance to targets and experiential context is consistent with the notion that VSPT in real-world settings is not a task that happens in isolation. For example, imagine sitting in the stands at a ball game waiting for a friend. Your friend is lost, but you can see him under the scoreboard. By phone, you might give him directions based on what you know he can currently see. The particular directions you give might be influenced by a wide variety of concerns—your friends known ability to mix up left and right, the urgency with which you want to get him to the seat before first pitch, whether you want him to pass the concession stand to grab refreshments, etc. In this example, the ability to relate to the friend's situation involves both the understanding of visual-spatial perspective (what the friend can see) but also the socially relevant situational factors (the friend's state, abilities, goals). Therefore, one's performance should be dependent on the interaction of spatial and social skills such that this socially relevant VSPT situation will benefit if social skills are strong but might be hindered if they are weaker.

This still leaves the broader question of why the specific judgments participants were asked to do in our VSPT task should be influenced by social skills when targets appear to convey agency. That is, one could imagine that the ballgame example could be accomplished by having the spatial reasoning done by a “purely” spatial computational process with social skills only entering at the point of deciding how to communicate that information to an agent. Our task only requires the judgment and not a tailored communication of the outcome, suggesting that the social influence may be operating throughout the process of perspective taking. Although our results are ambivalent with respect to how the presence of an agent-like target might be altering the underlying computations, we offer some speculation about how this interaction might come about. One possibility is that VSPT involves first essentially embodying the target of the potential perspective one is attempting to assess (i.e., one is attempting to assume the target's position in order to see its viewpoint). As may be the case with spontaneous perspective taking (Tversky and Hard, 2009), a potential agent as a target may automatically induce one to consider the target's social/personality attributes. One's comfort level in understanding or appreciating these attributes may then serve to gate how readily the perspective can be assumed. For example, an individual who is more socially savvy might be able to more readily recognize the utility of a potential agent through efficient assessment of relevant attributes (e.g., the eyes on the fashion doll means she might have the ability to see) and dismissal of irrelevant and unknown attributes (e.g., she is happy and has good fashion sense), whereas an individual with less social savvy might experience inhibition in trying to take the perspective of a potential agent because he/she cannot apprehend the attributes as readily and choose those that would facilitate embodiment. By contrast, when the perspective taking involves an object as the target, there are no obvious social attributes, so one's social savvy will neither hurt nor help, as it is irrelevant.

In this working model, we do not propose different mechanisms or processes for VSPT with agents vs. objects. Instead, we are suggesting that there is a common spatial component irrespective of the target, which is consistent with the observed correlations between the different versions of the VSPT task (targets with and without agency). However, when there is an agent, social skills may act as a gateway for the initial step of embodying the target. This proposed role suggests that we will still see individual differences in the spatial aspects of the tasks, regardless of target type, but we will have additional variability due to social skills when the target is a potential agent. Whether the proposed model above or an alternative framework ultimately captures the interaction of social and spatial skills, this work motivates a deeper question: what are the advantages of having a system that is generally sensitive to the presence of agents for a seemingly spatial task given that this sensitivity can benefit some individuals and hinder others relative to non-social conditions? Is this driven by the folk wisdom that humans are simply social beings? These are open questions and ones not readily addressed empirically, but they provide fodder for thinking critically about the interactive nature of human cognition.

The overarching goal of this project was to deepen the exploration of factors that determine whether and when VSPT might be sensitive to the influence of social skills. Taken together, the results suggest that the social influence on VSPT is mediated by a complex relationship that includes the task, the target, and the context in which the target is perceived. Future studies may continue to elaborate on the various boundary conditions that evoke agency or take it away, but the broader message from our work and similar studies is the importance of thinking beyond the bounds of a single domain to explain the complexity of human behavior.

### Conflict of interest statement

The authors declare that the research was conducted in the absence of any commercial or financial relationships that could be construed as a potential conflict of interest.

## Footnotes

^1^ One important note on social skills in this context is that the relevant dimension is likely to be one's understanding and appreciation of social attributes and situations rather than social-seeking behavior or extroversion. Throughout, we use this broader definition, suggesting a form of social intelligence or savvy rather than more strictly whether someone engages in more or less social activity.

^2^ This question was intentionally vague to limit potential bias in the language that might influence how the participant should interpret the task and the target. Using language such as “Who sees this… ?” might affect interpretation of agency (i.e., “who” denotes a being; “seeing” is a property of an agent). We also acknowledge that this wording does not explicitly tell the participant to mentally visualize the space. As such, it is possible that some participants engaged more or less visual imagery as opposed to other forms of spatial reasoning. However, we have no reason to suspect that this same variability in strategy does not hold for all VSPT tasks.

^3^ Consistent with previous data, response latency had no significant correlations (strongest was *r* = +0.1).
